# The correlation between different types of negative life events and the mental health status of Han ethnic adolescents in Sichuan Province

**DOI:** 10.3389/fpsyt.2026.1743626

**Published:** 2026-02-19

**Authors:** Shoukang Zou, Yangling Li, Wenli Tan, Meijiang Jin, Maojia Ran, Zhujun Wang, Hang Zhang, Hanmei Xu, Yuanmei Tao, Xian Tang, Ping Xiong, Huiping Huang, Ying Huang, Ling Li, Wenjuan Yang, Hongping Zeng, Gui Liu, Xiaosu Shen, Hongqin Zhao, Ying Chen, Kangling Yao, Jingyi Zhao, Wenwen Han, Jingmiao Zhou, Jianmin Hou, Shikun Peng, Yadan Wang, Yunzhen Yang, Yi Feng, Lin Chen, Xiting Yang, Shuangshuang Li, Xue Luo, Yan Wang, Li Yin

**Affiliations:** 1 Mental Health Center, West China Hospital of Sichuan University, Chengdu, Sichuan, China; 2 Department of Gastroenterology, West China Hospital of Sichuan University, Chengdu, Sichuan, China; 3 Chengdu Research Institute of Education Science, Chengdu, Sichuan, China; 4 Center for Disease Control and Prevention, Chengdu, Sichuan, China; 5 Chengdu Engineering Technical Vocational School, Chengdu, Sichuan, China; 6 Sichuan Bright Foreign Language School, Emeishan, Sichuan, China; 7 Chengdu Shishi Jincheng Foreign Language School, Chengdu, Sichuan, China; 8 Sichuan Chengdu Zhonghe Vocational High School, Chengdu, Sichuan, China; 9 Chengdu Eldo Primary School, Chengdu, Sichuan, China; 10 Majiahe Primary School of Chengdu, Chengdu, Sichuan, China; 11 Chengdu Huaxi Primary School, Chengdu, Sichuan, China; 12 Shude Xiejin High School, Chengdu, Sichuan, China; 13 Chengdu Wuhou Experimental Middle School Primary School, Chengdu, Sichuan, China; 14 Yinxing Primary School, Chengdu, Sichuan, China; 15 Southwest Jiaotong University Affiliated Middle School, Chengdu, Sichuan, China; 16 Tianfu Number 4 High School, Chengdu, Sichuan, China; 17 Chengdu Primary School Affiliated To Beijing International Studies University, Chengdu, Sichuan, China; 18 Chengdu Shuangqing Primary School, Chengdu, Sichuan, China; 19 Chengdu Shayan Primary School, Chengdu, Sichuan, China; 20 Chengdu Xin Qiao Primary School, Chengdu, Sichuan, China; 21 Institute for System Genetics, Frontiers Science Center for Disease-related Molecular Network, Chengdu, Sichuan, China; 22 Sichuan Clinical Medical Research Center for Mental Disorder, Chengdu, Sichuan, China

**Keywords:** Han ethnic adolescents, influencing factors, mediation effect, mental health status, negative life events

## Abstract

**Objectives:**

Although many studies have shown a significant association between negative life events and mental health problems among adolescents, few studies have explored whether there are differences in the impact of different types of negative life events on mental health problems among adolescents. We hope to further explore and analyze this issue through a cross-sectional study of Han ethnic adolescents in Sichuan Province.

**Methods:**

Cluster sampling was adopted to analyze anxiety symptoms, depressive symptoms and suicidal ideation and their influencing factors among 9,982 students from 4 middle schools in Sichuan Province, western China. We used the Adolescent Life Events Scale to evaluate the negative life events experienced by adolescent students in the past year. we use the Generalized Structural Equation Model (GSEM) to calculate the chain mediation effect between the negative life events and mental health status. The influence of different negative life events on different mental health problems was explored through binary logistic regression.

**Results:**

A higher level of core self-evaluation was a protective factor for anxiety symptoms, depressive symptoms and suicidal ideation among adolescent students. Adequate sleep was a protective factor for depressive symptoms and suicidal ideation among adolescent students. Vocational high school education and sufficient exercise were the protective factors for anxiety symptoms among adolescent students. After controlling for gender, age, school type, and exercise, sleep and core self-evaluation exhibited a significant chain mediating effect in the relationship between negative life events and anxiety symptoms/depressive symptoms (effect value: 0.0131, p<0.001, 95% CI: 0.0086- 0.0177; effect value:0.0116, p<0.001, 95% CI: 0.0076- 0.0155). For negative life events, interpersonal stress and adaptation problem were risk factors for anxiety symptoms, depressive symptoms and suicidal ideation among adolescent students (OR = 1.179, p<0.001, 95% CI 1.149–1.209; OR = 1.187, p<0.001, 95% CI 1.153–1.222; OR = 1.074, p<0.001, 95% CI 1.048–1.100; OR = 1.163, p<0.001, 95% CI 1.128–1.199; OR = 1.229, p<0.001, 95% CI 1.188–1.271; OR = 1.044, p=0.002, 95% CI 1.016–1.073). Loss was a risk factor for depressive symptoms and suicidal ideation among adolescent students (OR = 1.050, p<0.001, 95% CI 1.022–1.079; OR = 1.030, p=0.009, 95% CI 1.008–1.054). Punishment was the risk factor for depressive symptoms among adolescent students (OR = 1.037, p=0.012, 95% CI 1.008–1.067). Academic stress was the risk factor for anxiety symptoms among adolescent students (OR = 1.077, p<0.001, 95% CI 1.051–1.104).

**Conclusion:**

Sleep and core self-evaluation are important mediating factors in the relationship between negative life events and mental health status. Interpersonal stress and adaptation problem in negative life events were the main risk factors for mental health problems among adolescent students. We expect to provide data to support the early prevention and intervention of mental health problems among adolescents. Special attention should be given to the mental health status of adolescent students with interpersonal relationship problems and maladaptation.

## 1 Introduction

In 2025, the World Health Organization (WHO) noted that one in seven adolescents aged 10–19 years worldwide had one type of mental disorder, accounting for 15% of the global disease burden in this age group (1). Among them, depression, anxiety and behavioral disorders were the main causes of illness and disability among adolescents, and suicide was the third leading cause of death among people aged 15–29 years (1). Meta-analysis revealed that the proportion of children and adolescents in Europe suffering from mental disorders reached 15.5%, among which the diseases with the highest prevalence included anxiety disorder and attention deficit hyperactivity disorder (ADHD) (2). A survey from three low-income countries revealed that the prevalence of mental disorders among teenagers in the past 12 months was 12.1% in Kenya, 5.5% in Indonesia and 3.3% in Vietnam (3). A cohort study from Canada revealed that depressive symptoms during childhood and adolescence were closely related to depressive symptoms and impaired psychosocial functions in early adulthood (4). The prevalence of mental health problems among adolescents is high, which can lead to serious consequences. This requires the active attention of the whole society and the strengthening of early prevention and intervention.

Identifying more modifiable influencing factors regarding adolescent mental health problems is conducive to the implementation of early prevention and intervention health policies and measures. A survey of middle school students from Anhui Province, China, revealed that the risk factors for mental health problems included girl and high school, whereas two-parent families were protective factors (5). A survey from Philadelphia revealed that risk factors for mental health among teenagers, such as depressive symptoms and suicide, included exposure to trauma, school bullying, substance abuse, fighting, and living in a high-pressure community (6). Research conducted by Eva Oberle revealed that participation in extracurricular activities was associated with lower levels of anxiety and depressive symptoms, and longer screen time (≥2 hours per day) was associated with higher levels of anxiety and depressive symptoms (7). A follow-up study from Sweden revealed that a high level of physical activity in early adolescence was associated with lower depressive symptoms three years later (8). Mina Shimizu conducted a longitudinal study of 199 teenagers and reported that sleep–wake problems in late childhood were associated with mental health issues such as depression and anxiety during adolescence (9). At present, many studies had shown that a higher level of core self-evaluation was associated with a lower level of depression in teenagers (10, 11). A cohort study from Oxford University revealed that a stronger learning atmosphere was associated with a lower level of depression and greater well-being (12).

Many studies showed that negative life events such as childhood abuse and school bullying were associated with poorer mental health problems among adolescents (13–15). A study from France revealed that victims of parental abuse or peer bullying had significantly increased risks of anxiety, depression and suicide (16). A survey in Shandong Province revealed that negative life events in the past year were associated with suicidal ideation among college freshmen. Among students without or with mild depression, negative life events related to interpersonal relationships were associated with suicidal ideation, among students with moderate to severe depression, negative life events related to health and adaptation were associated with suicidal ideation (17). One meta-analysis revealed that academic stress was correlated with at least one mental health problem among adolescents (18). A survey of more than 200,000 teenagers in South Korea revealed that poor interpersonal relationships during stressful life events were the strongest risk factor associated with depression and suicide (19).

Although many studies have shown an association between negative life events and mental health problems in adolescents, the psychological mechanisms underlying the relationship between negative life events and mental health also need to be further clarified, and few studies have compared the relationships between different types of negative life events and mental health problems in adolescents. Therefore, this study explores the psychological mechanisms underlying the relationship between negative life events and mental health status and the correlations between different types of negative life events and anxiety symptoms, depressive symptoms, and suicidal ideation among middle school students in Sichuan Province, China. This study is intended to provide a basis for the early prevention and intervention of adolescent mental health problems in the future.

## 2 Methods

### 2.1 Participants

From October 2020 to March 2021, via the cluster sampling method, electronic questionnaires were distributed to all students aged 11–18 years in four schools in Chengdu city (1 junior high school and 2 vocational middle schools) and Leshan city (1 middle school including junior senior) through the teachers of each school. Sample size calculation: We used depressive symptoms in adolescents with a higher incidence rate among the outcome variables as parameters, and the statistical power was 0.8. We calculated the sample size on the basis of a 25% prevalence rate of depressive symptoms among adolescents (20, 21), with a margin of error of 0.02 and a type I error of 0.05. By substituting the sampling survey formula for calculating the rate via the PASS software, we calculated the sample size n=1849. It was estimated that 20% of the students might be lost or not cooperative. The final sample size was determined to be 2312 cases. The actual sample of 9,982 cases was larger than the theoretical sample, so the entire sample was representative.

### 2.2 Ethical approval and informed consent

The study was approved by the Ethics Committee of the West China Hospital of Sichuan University. The legal guardians (parents) of minors were informed of the significance, confidentiality and safety of the research through the electronic informed consent form and signed the electronic informed consent form. In accordance with the principles of equality, voluntariness and cooperation, investigations will be conducted only on those who have agreed and signed the informed consent form.

### 2.3 Investigation tools

The Core Self-Evaluations Scale (CSES) uses the Chinese version compiled by Judge and translated and revised by Jianzheng Du to evaluate the core self-evaluations of adolescents. This scale consists of 10 items and is scored on a 5-point scale, with points from 1 to 5 indicating “completely disagree” to “completely agree”, respectively (22, 23). The Center for Epidemiologic Studies Depression Scale (CES-D) has a total of 20 items and is a self-assessment scale. The participants were required to rate their symptoms on a 4-point scale (1–4 points) on the basis of the frequency of symptom occurrence, with a focus on evaluating depressive emotions or moods within a week. The structure of the CES-D can be divided into four factors: (1) Somatic symptoms; (2) Negative emotions; (3) Positive emotions; and (4) Interpersonal relationships. The higher the score is, the greater the degree of depression (24, 25). We selected item 9 on suicidal ideation in the Beck Depression Inventory-II to assess students’ suicidal ideation, which was divided into four levels. A score of 0 indicates no suicidal thoughts at all, and a score of 1 indicated having suicidal thoughts but not taking action. A score of 2 indicates a desire to commit suicide, and a score of 3 indicates that one would commit suicide if given the chance (26, 27). The Screen for Child Anxiety Related Emotional Disorders (SCARED) is a screening form for children’s anxiety symptoms developed by Birmaher. The score is calculated on three levels (0–2 points), with 0 points indicating that there is no such problem. A score of 1 indicates that the problem sometimes exists, and a score of 2 indicates that it occurs frequently (28, 29). The Adolescent Self-Rating Life Events Check List (ASLEC) was developed by Xiancheng Liu in China. It consists of 27 items and assesses negative life events that occurred in life over the past year. The score is calculated on five levels (1 to 5 points). A score of 1 indicates that the event has not occurred or has no impact. A score of 2 indicates a mild impact. A score of 3 indicates a moderate impact. A score of 4 indicates a severe impact. A score of 5 indicates an extremely severe impact. Xiuhong Xin subdivided the ASLEC into five factors: the punishment factor, the loss factor, the interpersonal stress factor, the academic stress factor and the adaptation factor (30, 31). A self-compiled questionnaire was used to collect the demographic data of the students, such as gender, age and ethnicity, as well as their sleep and exercise conditions. Regarding the average sleep status per night, the possible options were”4 hours or less”, “5 hours”, “6 hours”, “7 hours”, “8 hours”, “9 hours”, “10 hours”, “11 hours” or “12 hours and more”. Regarding the average daily duration of moderate- or high-intensity exercise, the possible options were “less than 1 hour”, “1 hour”, “2 hours”, “3 hours”, “4 hours” and “5 hours or longer”. If the students were 13 years old or younger, we defined adequate sleep as at least 9 hours per day; if the students were 14 years old or older, we defined adequate sleep as at least 8 hours per day (32, 33). We defined adequate exercise as at least one hour of moderate or vigorous exercise every day (34).

### 2.4 Statistical analysis

All the data analyses were conducted via SPSS 23.0(IBM, Armonk, NY, USA) and Stata version 17.0(StataCorp, College Station, Texas, USA), which mainly employed descriptive analysis, correlation analysis, and binary logistic regression analysis in SPSS, and we use the Generalized Structural Equation Model (GSEM) to calculate the chain mediation effect between the negative life events and mental health status in Stata. For the qualitative data, the proportion of the variable in the entire sample was described as a percentage, and the correlation analysis between the variable and the categorical outcome variable was conducted through the chi-square test. For the quantitative data, X ± S was used to describe the average value, and the correlation analysis between this variable and the categorical outcome variable was conducted through the t test. First, we initially screened the correlations between gender, age, ethnicity, school type, whether sleep was adequate, whether exercise was adequate, the score of the CSES, the score of the ASLEC and the mental health status (anxiety symptoms, depressive symptoms, suicidal ideation) of adolescent students through chi-square tests or t tests (p<0.05 was considered to indicate a correlation). We used negative life events as the independent variable, and the scores of anxiety or depressive symptom scales as the dependent variable in the GSEM. We also controlled for gender, age, school type, and exercise. We explored whether sleep and core self-evaluation acted as chain mediators to ultimately influence anxiety and depressive symptoms. Binary logistic regression was subsequently used to evaluate the specific impact of the screened relevant influencing factors on the mental health status of adolescent students. A bilateral P value <0.05 was considered statistically significant.

## 3 Result

### 3.1 Demographic data and score of related scales

A total of 11,327 questionnaires were distributed, and 9,982 valid questionnaires were retrieved, with an effective recovery rate of 88.1%. Among them, 5,439 (54.5%) were male, with an average age of 15.28 ± 1.64 years (age range 11–18 years). Among the ethnic groups, there were 9,616 (96.3%) Han students, followed by 174 (1.7%) Tibetan students, 112 (1.1%) Yi students, and a total of 80 (0.8%) students were from other ethnic groups. There were 4,293 (43%) students in ordinary middle schools. There were 5,029 (50.4%) students who did not have adequate sleep and 2,203 (22.1%) students who did not have adequate exercise. The average score of the CSES was 34.90 ± 7.75 points. The average ASLEC score was 37.61 ± 13.01 points. The average score of the SCARED was 18.58 ± 15.41 points. The average score of the CES-D was 16.08 ± 9.57 points. A total of 2,822 students had suicidal ideation (28.3%, and those with a score of 1, 2, or 3 on the suicidal ideation item on the BDI-II scale were judged as having suicidal ideation). There were 2,998 students with depressive symptoms (30%, with a CES-D scale score of 20 or above). There were 3,452 students with anxiety symptoms (34.6%, with a SCARED scale score of 23 or above). The proportions of students with suicidal ideation, depressive symptoms, and anxiety symptoms were described in our previous article (35). (See **Table 1** for details).

**Table 1:** Demographic data and related scale scores (number of students, % / X±S).

Item	Distribution
Gender	
Male	5439(54.5%)
Female	4543(45.5%)
Age	15.28 ± 1.64
Ethnic nationality	
Han	9616(96.3%)
Tibetan	174(1.7%)
Yi	112(1.1%)
Others	80(0.8%)
School type	
Ordinary middle school	4293(43%)
Vocational high school	5689(57%)
Whether sleep is adequate?	
No	5029(50.4%)
Yes	4953(49.6%)
Whether exercise is adequate?	
No	2203(22.1%)
Yes	7779(77.9%)
Scale Score	
CSES score	34.90 ± 7.75
ASLEC score	37.61 ± 13.01
SCARED score	18.58 ± 15.41
CES-D score	16.08 ± 9.57
Suicidal ideation	
0	7160(71.7%)
1	2395(24%)
2	305(3.1%)
3	122(1.2%)

ASLEC, Adolescent Self-Rating Life Events Checklist; CES-D, Center for Epidemiological Survey Depression Scale; CSES, Core Self- Evaluation Scale; SCARED, Screen for Child Anxiety-Related Emotional Disorders.

### 3.2 Psychological mechanisms underlying the impact of negative life events on mental health status

#### 3.2.1 Psychological mechanisms underlying the impact of negative life events on anxiety

**Figure 1:**
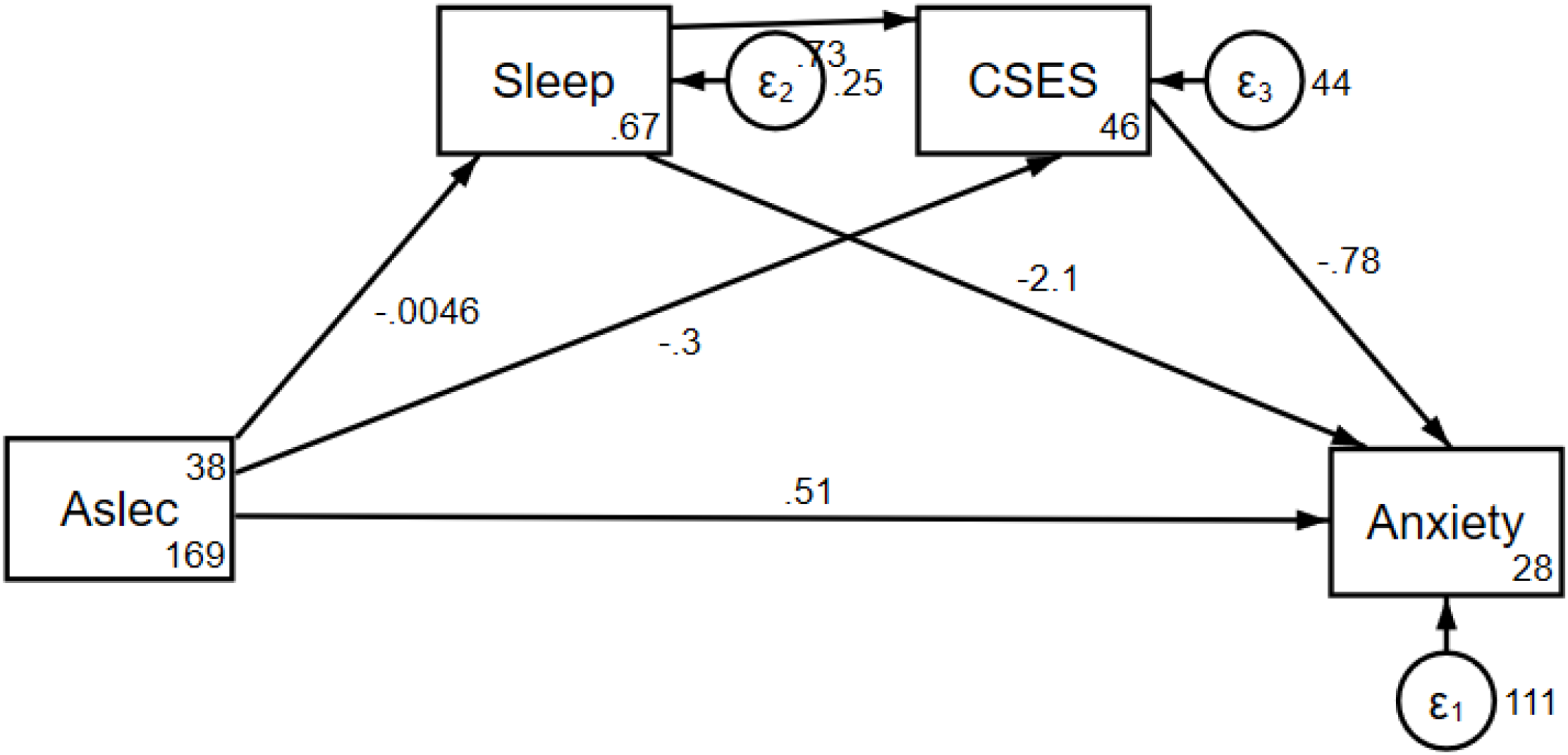
The chain mediating effect path diagram of sleep and CSES in the relationship between negative life events and anxiety symptoms. Aslec, Adolescent Self-Rating Life Events Checklist; CSES, Core Self- Evaluation Scale.

We use Generalized Structural Equation Model (GSEM) to calculate the chain mediation effect. After controlling for gender, age, school type, and exercise, sleep and core self-evaluation exhibited a significant chain mediating effect in the relationship between negative life events and anxiety symptoms (effect value: 0.0131, p<0.001, 95% CI: 0.0086- 0.0177). (See **Figure 1**; **Appendix Table 1** for details).

#### 3.2.2 Psychological mechanisms underlying the impact of negative life events on depression

**Figure 2:**
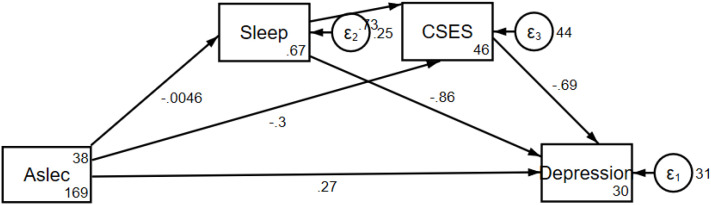
The chain mediating effect path diagram of sleep and CSES in the relationship between negative life events and depressive symptoms. Aslec, Adolescent Self-Rating Life Events Checklist; CSES, Core Self- Evaluation Scale.

We use Generalized Structural Equation Model (GSEM) to calculate the chain mediation effect. After controlling for gender, age, school type, and exercise, sleep and core self-evaluation exhibited a significant chain mediating effect in the relationship between negative life events and depressive symptoms (effect value:0.0116, p<0.001, 95% CI: 0.0076- 0.0155). (See **Figure 2**; **Appendix Table 2** for details).

### 3.3 Correlation analysis

#### 3.3.1 Relevant influencing factors of anxiety symptoms

In this study, binary logistic regression was used to evaluate the effects of the initial screened variables, including age, gender, school type, whether sleep is adequate, whether exercise is adequate, CSES score, and five factors in the ASLEC (punishment factor, loss factor, interpersonal stress factor, academic stress factor, and adaptation factor), on whether anxiety symptoms occurred in the students. The final logistic regression model was statistically significant (x2 = 4849.592, p<0.001). This model could correctly classify 81.9% of the research subjects. The sensitivity of the model was 66.6%, and the specificity was 90.0%. In this investigation, girl gender was a risk factor for anxiety symptoms (OR = 1.819, p<0.001, 95% CI 1.625–2.037). No significant association between age and anxiety symptoms was found (OR = 0.960, p=0.066, 95% CI 0.918–1.003). Compared with ordinary middle school, vocational high school was a protective factor for anxiety symptoms (OR = 0.680, p<0.001, 95% CI 0.578–0.800). No significant association was found between whether sleep is adequate and anxiety symptoms (OR = 0.930, p=0.235, 95% CI 0.825–1.048). Adequate exercise was a protective factor against anxiety symptoms (OR = 0.862, p=0.035; 95% CI 0.751–0.990). A higher CSES score was a protective factor for anxiety symptoms (OR = 0.854, p<0.001, 95% CI 0.845-0.863). No significant correlation was found between the score of the punishment factor and the loss factor in the ASLEC and the anxiety symptoms of the students (OR = 1.012, p=0.367, 95% CI 0.986–1.038; OR = 1.020, p=0.111, 95% CI 0.995–1.045). The interpersonal stress factor, the academic stress factor and adaptation factor were risk factors for anxiety symptoms (OR = 1.179, p<0.001, 95% CI 1.149–1.209; OR = 1.077, p<0.001, 95% CI 1.051–1.104; OR = 1.163, p<0.001, 95% CI 1.128–1.199) (see **Table 2** for details).

**Table 2:** Related influencing factors of anxiety symptoms (binary logistic regression analysis).

Variables	B	SE	Wald	OR (95%CI)	P
Gender (Female/Male)	0.598	0.058	107.743	1.819(1.625-2.037)	<0.001
Age	-0.041	0.022	3.380	0.960(0.918-1.003)	=0.066
School type	-0.386	0.083	21.519	0.680(0.578-0.800)	<0.001
Whether sleep is adequate (Yes/No)	-0.072	0.061	1.408	0.930(0.825-1.048)	=0.235
Whether exercise is adequate (Yes/No)	-0.148	0.070	4.440	0.862(0.751-0.990)	=0.035
CSES score	-0.158	0.005	853.385	0.854(0.845-0.863)	<0.001
ASLEC score					
Punishment factor	0.012	0.013	0.815	1.012(0.986-1.038)	=0.367
Loss factor	0.020	0.012	2.544	1.020(0.995-1.045)	=0.111
Interpersonal stress factor	0.164	0.013	159.877	1.179(1.149-1.209)	<0.001
Academic stress factor	0.074	0.013	35.135	1.077(1.051-1.104)	<0.001
Adaptation factor	0.151	0.015	95.883	1.163(1.128-1.199)	<0.001

ASLEC, Adolescent Self-Rating Life Events Checklist; CSES, Core Self- Evaluation Scale.

#### 3.3.2 Relevant influencing factors of depressive symptoms

In this study, binary logistic regression was used to evaluate the effects of the initial screened variables, including age, gender, school type, whether sleep is adequate, whether exercise is adequate, CSES score, and five factors in the ASLEC, on whether depressive symptoms occurred in the students. The final logistic regression model was statistically significant (x2 = 6178.577, p<0.001). This model could correctly classify 86.4% of the research subjects. The sensitivity of the model was 71.3%, and the specificity was 92.8%. No significant associations between gender or age and depressive symptoms were found in this survey (OR = 1.055, p=0.427, 95% CI 0.924–1.204; OR = 1.049, p=0.068, 95% CI 0.996–1.105). No significant influence of school type on students’ depressive symptoms was found (OR = 0.873, p=0.162; 95% CI 0.721–1.056). Adequate sleep was a protective factor for depressive symptoms (OR = 0.786, p=0.001, 95% CI 0.684–0.904). However, whether exercise is adequate did not significantly affect the depressive symptoms of the students (OR = 0.905, p=0.225, 95% CI 0.769–1.064. A higher CSES score was a protective factor for depressive symptoms (OR = 0.750, p<0.001, 95% CI 0.738–0.762). No significant correlation was found between the score of the academic stress factor in the ASLEC and the depressive symptoms of the students (OR = 1.000, p=0.991; 95% CI 0.972–1.029). Among the ASLEC, the punishment factor, loss factor, interpersonal stress factor, and adaptation factor were all risk factors for depressive symptoms (OR = 1.037, p=0.012, 95% CI 1.008–1.067; OR = 1.050, p<0.001, 95% CI 1.022–1.079; OR = 1.187, p<0.001, 95% CI 1.153–1.222; OR = 1.229, p<0.001, 95% CI 1.188–1.271) (see **Table 3** for details).

**Table 3:** Related influencing factors of depressive symptoms (binary logistic regression analysis).

Variables	B	SE	Wald	OR (95%CI)	P
Gender (Female/Male)	0.054	0.068	0.630	1.055(0.924-1.204)	=0.427
Age	0.048	0.026	3.330	1.049(0.996-1.105)	=0.068
School type	-0.136	0.097	1.955	0.873(0.721-1.056)	=0.162
Whether sleep is adequate (Yes/No)	-0.240	0.071	11.384	0.786(0.684-0.904)	=0.001
Whether exercise is adequate (Yes/No)	-0.100	0.083	1.475	0.905(0.769-1.064)	=0.225
CSES score	-0.288	0.008	1248.294	0.750(0.738-0.762)	<0.001
ASLEC score					
Punishment factor	0.037	0.015	6.339	1.037(1.008-1.067)	=0.012
Loss factor	0.049	0.014	12.697	1.050(1.022-1.079)	<0.001
Interpersonal stress factor	0.171	0.015	136.193	1.187(1.153-1.222)	<0.001
Academic stress factor	<0.001	0.014	<0.001	1.000(0.972-1.029)	=0.991
Adaptation factor	0.206	0.017	141.168	1.229(1.188-1.271)	<0.001

ASLEC, Adolescent Self-Rating Life Events Checklist; CSES, Core Self- Evaluation Scale.

#### 3.3.3 Relevant influencing factors of suicidal ideation

In this study, binary logistic regression was used to evaluate the effects of the initial screened variables, including age, gender, school type, whether sleep is adequate, whether exercise is adequate, the CSES score, the SCARED score, the CES-D score and five factors in the ASLEC, on whether suicidal ideation occurred in the students. The final logistic regression model was statistically significant (x2 = 3588.038, p<0.001). This model could correctly classify 81.7% of the research subjects. The sensitivity of the model was 53.8%, and the specificity was 92.7%. In this investigation, girl was a risk factor for suicidal ideation (OR = 1.474, p<0.001, 95% CI 1.317–1.651). Older age was a protective factor for suicidal ideation (OR = 0.908, p<0.001, 95% CI 0.869–0.949). No significant influence of school type on students’ suicidal ideation was found (OR = 1.086, p=0.314; 95% CI 0.924–1.277). Adequate sleep was a protective factor for suicidal ideation (OR = 0.829, p=0.002, 95% CI 0.737–0.933). However, whether exercise is adequate did not significantly affect students’ suicidal ideation (OR = 0.969, p=0.649, 95% CI 0.846–1.110),. A higher CSES score was a protective factor for suicidal ideation (OR = 0.944, p<0.001, 95% CI 0.933–0.955). A higher CES-D score was a risk factor for suicidal ideation (OR = 1.078, p<0.001, 95% CI 1.066–1.090); a higher SCARED score was also a risk factor for suicidal ideation (OR = 1.011, p<0.001, 95% CI 1.006–1.017). In the ASLEC, no significant correlation was found between the punishment factor or the academic stress factor and suicidal ideation(OR = 1.013, p=0.276, 95% CI 0.990–1.036; OR = 1.023, p=0.063, 95% CI 0.999–1.047), the loss factor, interpersonal stress factor and adaptation factor were all risk factors for suicidal ideation (OR = 1.030, p=0.009, 95% CI 1.008–1.054; OR = 1.074, p<0.001, 95% CI 1.048–1.100; OR = 1.044, p=0.002, 95% CI 1.016–1.073) (see **Table 4** for details).

**Table 4:** Related influencing factors of suicidal ideation (binary logistic regression analysis).

Variables	B	SE	Wald	OR (95%CI)	P
Gender (Female/Male)	0.388	0.058	45.231	1.474(1.317-1.651)	<0.001
Age	-0.096	0.022	18.539	0.908(0.869-0.949)	<0.001
School type	0.083	0.082	1.012	1.086(0.924-1.277)	=0.314
Whether sleep is adequate (Yes/No)	-0.187	0.060	9.670	0.829(0.737-0.933)	=0.002
Whether exercise is adequate (Yes/No)	-0.032	0.069	0.207	0.969(0.846-1.110)	=0.649
CSES score	-0.058	0.006	90.252	0.944(0.933-0.955)	<0.001
CES-D score	0.075	0.006	177.449	1.078(1.066-1.090)	<0.001
SCARED score	0.011	0.003	16.737	1.011(1.006-1.017)	<0.001
ASLEC score					
Punishment factor	0.013	0.012	1.189	1.013(0.990-1.036)	0.276
Loss factor	0.030	0.011	6.864	1.030(1.008-1.054)	=0.009
Interpersonal stress factor	0.071	0.012	33.405	1.074(1.048-1.100)	<0.001
Academic stress factor	0.022	0.012	3.469	1.023(0.999-1.047)	0.063
Adaptation factor	0.043	0.014	9.791	1.044(1.016-1.073)	0.002

ASLEC, Adolescent Self-Rating Life Events Checklist; CES-D, Center for Epidemiological Survey Depression Scale; CSES, Core Self- Evaluation Scale; SCARED, Screen for Child Anxiety-Related Emotional Disorders.

## 4 Discussion

This study revealed that a higher level of core self-evaluations was a protective factor for anxiety symptoms, depressive symptoms and suicidal ideation among adolescent students. Adequate sleep was a protective factor for anxiety symptoms and suicidal ideation, and vocational high school and adequate exercise were protective factors for anxiety symptoms among adolescent students. Interpersonal stress-related and adaptation-related negative life events were risk factors for anxiety symptoms, depressive symptoms and suicidal ideation among adolescent students. Negative life events related to loss were risk factors for depressive symptoms and suicidal ideation, negative life events related to punishment were only risk factors for depressive symptoms, and negative life events related to academic pressure were only risk factors for anxiety symptoms among adolescent students.

Similar to the results of this study, a cohort study from the American Adolescent Brain Cognitive Development (ABCD) database revealed that during the transition from childhood to adolescence, sleep problems were associated with emotional and behavioral problems (36). Michael Gradisar reported that sleep problems made teenagers more prone to depression through circadian rhythm delay, limited sleep time, and repetitive negative thinking while trying to fall asleep (37). Through an 8-year follow-up study in a cohort of adolescents, Finnish researchers reported that higher levels of physical health and cardiopulmonary health during childhood and adolescence were associated with better cognitive abilities and improved mental health (38). In our previous study, we reported that a higher level of core self-evaluations was a protective factor for adolescent suicidal ideation and played a partial mediating role in the relationship between depressive symptoms and suicidal ideation (35). We have found that the vocational high school type is a protective factor against anxiety symptoms among adolescents. This might be because students from ordinary middle schools in Sichuan Province face greater academic pressure to enter higher education.

A cohort study of children and adolescents from Columbia University revealed that young women with more childhood adversity had a higher rate of suicidal ideation, and childhood adversity was associated with attempted suicide throughout life regardless of gender (39). A survey conducted in Stockholm revealed that the proportion of mental health problems among boys who had experienced school bullying was four times higher than that among boys who had not experienced school bullying, and the corresponding proportion was 2.4 times higher among girls (14). Shay Arnon et al. found through an analysis of the ABCD database that adolescent students who experienced cyberbullying were more likely to have suicidal tendencies (40). Researchers from Duke University conducted a 21-year follow-up study and reported that traumatic events and recent stressful events were significantly associated with anxiety and depressive symptoms in children, adolescents, and early adulthood, and the effects of traumatic events and recent stressful events were similar (41).

In this cross-sectional survey, after conducting a mediation effect analysis using the generalized structural equation model, we found that sleep and core self-evaluation have a chain mediating effect in the relationship between negative life events and anxiety/depression symptoms. This indicates that negative life events not only directly cause anxiety/depression symptoms but also indirectly affect the anxiety/depression symptoms of adolescents through sleep and core self-evaluation. We verified and expanded the psychological mechanism previously established by our team regarding the relationship between negative life events and depressive symptoms among the Yi ethnic youth population in Liangshan Prefecture (42). In the early intervention and prevention of emotional symptoms in this population, it is necessary to pay attention to the sleep and core self-evaluation of adolescents.

This study revealed that different types of negative life events had different effects on mental health problems among adolescents. Interpersonal stress-related and adaptive-related (Significant changes in lifestyle/Disliking going to school/Breakup/Being distant from family members/Tense relationship with teachers) negative life events were common risk factors for three mental health problems among adolescent students: anxiety symptoms, depressive symptoms, and suicidal ideation, whereas academic stress-related negative life events were risk factors for anxiety symptoms. Negative life events related to punishment were risk factors for depressive symptoms, whereas negative life events related to loss were risk factors for both depressive symptoms and suicidal ideation. A follow-up study from Norway revealed that academic stress was significantly associated with psychological distress, such as anxiety and depressive symptoms, in adolescents three years later and that psychological distress also affected academic stress in the future (43). Yilin Hua and colleagues from Sun Yat-sen University further discovered that methylation of the NR3C1 gene significantly mediated the association between academic stress and anxiety symptoms in Chinese adolescents (44). One study in Zhejiang Province revealed that sleep problems play a mediating role in the association between adolescents’ exposure to domestic violence and depressive symptoms (45). A large-sample cross-sectional study of over 260,000 adolescents from Deyang city, Sichuan Province, revealed a strong association between traumatic events related to interpersonal violence and a relatively high suicide rate, among which physical abuse showed the strongest correlation (46). Cathryn Rodway and colleagues, after reviewing all 10–19-year-old teenagers (a total of 595) who died by suicide in the UK from 2014–2016, reported that teenagers with bereavement accounted for a quarter of the total number. Compared with teenagers without bereavement, teenagers with bereavement were more likely to commit suicide (47). Scholars from the University of California reported in a six-year follow-up study of more than 600 teenagers in Los Angeles and Chicago that the associations between chronic interpersonal stress and fear and anhedonia were mediated by irritability (48). Researchers from the University of North Carolina induced interpersonal stress in teenage girls through an improved version of the Trier Social Stress Test (TSST) and subsequently reported that the sluggish reactivity of cellular inflammatory factors (IL-1β, IL-6, and TNF-α) increased the risk of suicide behavior in individuals with high interpersonal stress in the subsequent 9 months (49). Different types of negative life events may affect the mental health status of adolescent students through genetics, inflammatory factors, brain functions, etc. However, most current studies are still limited to cross-sectional studies, and further exploration of their possible mechanisms is needed in the future.

Our research also has certain limitations. First, this study was a cross-sectional study conducted through a self-rating scale. Causal inference could not be made. The self-rating scale could only conduct a preliminary assessment of related symptoms and could not represent the diagnosis of depression and anxiety disorders, and it was also difficult to avoid recall bias. Second, all the students in this study were middle school students from Sichuan Province, and their ethnic group was mainly Han. This result is difficult to extend to other regions and ethnic groups. Third, the influencing factors of mental health problems are multifaceted and include genetics, the psychosocial environment, inflammation, personality traits, etc. This study focused mainly on the psychosocial environment and had certain limitations (50, 51). Fourth, the ASLEC we adopted assessed the negative life events that occurred in adolescents in the past year, ignoring the impact of previous negative life events on current mental health status, such as the influence of past childhood abuse and major traumatic events. Because the early childhood adverse experiences of teenagers have not been investigated, it may lead to an overestimation of the current related mental health outcomes, as some of these adverse outcomes are caused by earlier adverse experiences.

Despite certain limitations, this study revealed that sleep and core self-evaluation were important mediating factors in the relationship between negative life events and mental health status, and explored the different effects of different types of negative life events on anxiety symptoms, depressive symptoms, and suicidal ideation among adolescent students in Sichuan Province, China, through a large-sample cross-sectional survey. Interpersonal stress and adaptation-related negative life events were the main risk factors for mental health problems among adolescent students. We expect to provide data support for the early prevention and intervention of mental health problems among adolescents, with particular attention given to the mental health status of adolescent students with interpersonal relationship problems and maladaptation.

## Data Availability

The raw data supporting the conclusions of this article will be made available by the authors, without undue reservation.
