# Supplementary material for: The correlation between different types of negative life events and the mental health status of Han ethnic adolescents in Sichuan Province
Source: Front Psychiatry. 2026 Feb 19;17:1743626. doi: 10.3389/fpsyt.2026.1743626 (PMC12961804; doi:10.3389/fpsyt.2026.1743626)
Supplement: Supplementary file 1 [file Table1.docx]

| Regression model | | | | | | |
| --- | --- | --- | --- | --- | --- | --- |
| Dependent variable | Independent variable | Coefficient | Std. err. | z | P>\|z\| | [95% conf. interval] |
| Sleep | Aslec | -0.0118505 | 0.0017106 | -6.93 | <0.001 | -0.0152033, -0.0084977 |
|  | Gender | -0.2078071 | 0.044141 | -4.71 | <0.001 | -0.2943219, -0.1212924 |
|  | Age | -0.0002822 | 0.0180883 | -0.02 | 0.988 | -0.0357346, 0.0351702 |
|  | SchoolType | 1.457979 | 0.0610182 | 23.89 | <0.001 | 1.338385, 1.577572 |
|  | Exercise | 0.5711891 | 0.0568927 | 10.04 | <0.001 | 0.4596815, 0.6826967 |
| CSES | Aslec | -0.3058341 | 0.0050306 | -60.79 | <0.001 | -0.3156939, -0.2959742 |
|  | Sleep | 1.423987 | 0.1405969 | 10.13 | <0.001 | 1.148422, 1.699552 |
|  | Gender | -1.827568 | 0.1309971 | -13.95 | <0.001 | -2.084318, -1.570818 |
|  | Age | 0.0250271 | 0.0522468 | 0.48 | 0.632 | -0.0773748, 0.1274289 |
|  | SchoolType | -2.807857 | 0.18627 | -15.07 | <0.001 | -3.17294, -2.442775 |
|  | Exercise | 1.160245 | 0.1670813 | 6.94 | <0.001 | 0.832772, 1.487719 |
| Anxiety syptoms | CSES | -0.7782034 | 0.0158906 | -48.97 | <0.001 | -0.8093485, -0.7470583 |
|  | Aslec | 0.4905645 | 0.0093492 | 52.47 | <0.001 | 0.4722404, 0.5088886 |
|  | Sleep | -0.5455917 | 0.2243602 | -2.43 | 0.015 | -0.9853296, -0.1058538 |
|  | Gender | 3.84001 | 0.209993 | 18.29 | <0.001 | 3.428432, 4.251589 |
|  | Age | -0.1865595 | 0.0829497 | -2.25 | 0.025 | -0.3491379, -0.0239812 |
|  | SchoolType | -2.765877 | 0.2990752 | -9.25 | <0.001 | -3.352053, -2.1797 |
|  | Exercise | -1.035583 | 0.2659035 | -3.89 | <0.001 | -1.556744, -0.5144213 |

Appendix Table 1: GSEM to identify mediation effects（anxiety symptoms）

Abbreviations: Aslec, Adolescent Self-Rating Life Events Checklist; CSES, Core Self- Evaluation Scale;

Appendix Table 2: GSEM to identify mediation effects（Depressive symptoms）

| Regression model | | | | | | |
| --- | --- | --- | --- | --- | --- | --- |
| Dependent variable | Independent variable | Coefficient | Std. err. | z | P>\|z\| | [95% conf. interval] |
| Sleep | Aslec | -0.0118505 | 0.0017106 | -6.93 | <0.001 | -0.0152033, -0.0084977 |
|  | Gender | -0.2078071 | 0.044141 | -4.71 | <0.001 | -0.2943219, -0.1212924 |
|  | Age | -0.0002822 | 0.0180883 | -0.02 | 0.988 | -0.0357346, 0.0351702 |
|  | SchoolType | 1.457979 | 0.0610182 | 23.89 | <0.001 | 1.338385, 1.577572 |
|  | Exercise | 0.5711891 | 0.0568927 | 10.04 | <0.001 | 0.4596815, 0.6826967 |
| CSES | Aslec | -0.3058341 | 0.0050306 | -60.79 | <0.001 | -0.3156939, -0.2959742 |
|  | Sleep | 1.423987 | 0.1405969 | 10.13 | <0.001 | 1.148422, 1.699552 |
|  | Gender | -1.827568 | 0.1309971 | -13.95 | <0.001 | -2.084318, -1.570818 |
|  | Age | 0.0250271 | 0.0522468 | 0.48 | 0.632 | -0.0773748, 0.1274289 |
|  | SchoolType | -2.807857 | 0.18627 | -15.07 | <0.001 | -3.17294, -2.442775 |
|  | Exercise | 1.160245 | 0.1670813 | 6.94 | <0.001 | 0.832772, 1.487719 |
| Depressive syptoms | CSES | -0.6853456 | 0.0085794 | -79.88 | <0.001 | -0.7021609, -0.6685304 |
|  | Aslec | 0.2700677 | 0.0050476 | 53.50 | <0.001 | 0.2601745, 0.2799609 |
|  | Sleep | -0.8383479 | 0.1211325 | -6.92 | <0.001 | -1.075763, -0.6009327 |
|  | Gender | -0.0303949 | 0.1133756 | -0.27 | 0.789 | -0.252607, 0.1918171 |
|  | Age | 0.0093054 | 0.0447847 | 0.21 | 0.835 | -0.0784709, 0.0970817 |
|  | SchoolType | 0.0821849 | 0.1614712 | 0.51 | 0.611 | -0.2342929, 0.3986627 |
|  | Exercise | -0.3864312 | 0.1435618 | -2.69 | 0.007 | -0.6678071, -0.1050552 |

Abbreviations: Aslec, Adolescent Self-Rating Life Events Checklist; CSES, Core Self- Evaluation Scale;
